# Production of synthetic rutile from tin ore beneficiation byproduct through preoxidation and reductive leaching in hydrochloric acid

**DOI:** 10.1038/s41598-022-13250-w

**Published:** 2022-05-31

**Authors:** M. R. Kurniawan, T. G. Imami, Z. T. Ichlas, T. Hidayat, M. Z. Mubarok

**Affiliations:** Department of Metallurgical Engineering, Faculty of Mining and Petroleum Engineering, Institut Teknologi Bandung, Bandung, West Java Indonesia

**Keywords:** Engineering, Chemical engineering

## Abstract

This paper examines the effectiveness of the method for producing synthetic rutile from ilmenite through pre-oxidation and reductive leaching of pre-oxidized ilmenite in hydrochloric acid. Thermodynamic simulation of the pre-oxidation of ilmenite concentrate was performed to evaluate the phases formed during the process as a function of temperature. The pre-oxidation experiments were performed at different temperatures between 700 and 1000 °C in a muffle furnace for 6 h. The optimum temperature of pre-oxidation was revealed to be at 700 °C where ilmenite transformed into hematite and rutile, which is in accordance with the result of the thermodynamic simulation. Series of the leaching experiments were carried out under variations of HCl concentration (5–8 M), leaching temperature (70–100 °C), solid/liquid ratio (1/5–1/20 g/mL), ilmenite ore particle size distribution, and duration of leaching (6–12 h). Taguchi method utilizing L16 orthogonal array was adopted in the leaching step to design and reduce the required number of experiments. Analysis of variance (ANOVA) indicated that the temperature and solid/liquid (S/L) ratio were the most influential leaching parameters for the dissolution of iron and titanium. The optimum conditions for maximising the dissolution of iron, while minimizing the dissolution of titanium were at a temperature of 80 °C, HCl of 6 M, S/L ratio of 1/20 g/mL, ore particle size distribution of 44–77 µm (-200 + 325 mesh), and leaching duration of 6 h. The leaching experiment conducted under these conditions resulted in iron extraction of 98.07% with co-extraction of titanium of 11.35%. The leach-residue contains 92.6% rutile, 2.9% hematite, and 2.5% cassiterite which can be classified as synthetic grade rutile.

## Introduction

The mineral cassiterite (SnO_2_), the primary source of tin metal, is often associated with ilmenite (FeTiO_3_) in nature. Hence, the processing of cassiterite often produces a by-product that has high contents of ilmenite, which is the primary source of titanium dioxide (TiO_2_) and titanium metal. Zglinicki et al.^1^ reported that the TiO_2_ concentration in the by-product of tin ore beneficiation in Bangka Island, Indonesia, can be as high as 51%. This suggests its potential as a resource for TiO_2_ production since the concentration is within the range of a commercial ilmenite concentrate, which is 40–65% TiO_2_.

The pigment industry consumes more than 90% of the global TiO_2_ production due to its high refractive index and inertness. The commercial production of TiO_2_ pigment has been carried out by two distinct methods, namely the sulphate and chloride process. The latter is considered cheaper since the hydrochloric acid is recycled and is considered cleaner since it produces lower volumes of waste and less invasive residues than the former^2, 3^. Unlike the former, however, the latter requires higher feed grades of about 90% TiO_2_^4, 5^. Therefore, an ilmenite concentrate needs to be upgraded before it can be fed into the chloride process. It is notable that the growing titanium metal industry also relies on such a high-grade feed material.

There are several processes to upgrade ilmenite for producing the so-called synthetic rutile, the mineral obtained through chemical alteration of ilmenite, as a feed for the chloride process. The commercial processes to produce synthetic rutile include the Becher process^6^, Benelite process^7^, Murso process^8^, and Austpac process^9^, which all involve roasting and leaching. In the Becher process, the ilmenite is oxidised with air to convert the iron in the ilmenite into hematite (Fe_2_O_3_), reduced with coal to convert the iron oxides into metallic iron and aerated in a large tank to re-oxidise the iron. The fine oxidised iron is precipitated in a form of slime and separated from the larger synthetic rutile particles. The residual iron oxides from the product are then leached with sulphuric acid and removed from the synthetic rutile. The Benelite process involves the reduction of ilmenite to convert the ferric iron into the ferrous state and hydrochloric acid is subsequently used to leach the iron. In the Murso process, the ilmenite is oxidised and then reduced in a fluidised bed to convert the ferric ion into the ferrous state and the product is then leached with hydrochloric acid. Similar to the Murso process, the Austpac process applies oxidation and reduction in a fluidised bed, but the two roasting steps are also aimed to selectively magnetise the ilmenite to allow the separation of the gangue mineral by magnetic separation. Hydrochloric acid leaching is used to remove iron and other impurities. The residue is then calcined and subjected to a magnetic separation step to obtain a high purity synthetic rutile.

These commercial processes involve a reduction step that is not only costly as the process needs to be performed at temperatures higher than 1000 °C but also environmentally undesirable as it generates abundant carbon dioxide due to the use of carbonaceous materials as reductants. Hence, there are many research efforts in recent years to minimise the thermochemical treatment steps in producing synthetic rutile. Mahmoud et al.^10^ and Lasheen^11^, for example, proposed the use of direct acid leaching with concentrated hydrochloric acid solutions in the presence of a reducing agent. The iron powder was used as a reductant in both studies and its addition not only increased the iron dissolution but also reduced the co-dissolution of the titanium. Lasheen^11^, however, showed that despite using highly concentrated (i.e., 12 M) of the acid, the total iron dissolution after 8 h of leaching at 90 °C was only 86%. The concentration of TiO_2_ in the synthetic rutile at the optimum condition was, therefore, relatively low at about 89% with about 7% of Fe_2_O_3_ remaining in the product. Similarly, the TiO_2_ concentration in the product obtained by Mahmoud et al.^10^ was also below 90%, which may still not suitable for the feed of the chloride process.

Vásquez and Molina^12^ showed that by oxidising the ilmenite at 700 °C for 6 h before leaching the iron, both the iron removal efficiency and the titanium recovery can be improved. This is because the pre-oxidation step converted the ilmenite into hematite and rutile with a layered configuration, as well as refractory magnetite, if present, into hematite. They reported that the subsequent reductive leaching of the pre-oxidised ilmenite can produce a residue of synthetic rutile with a TiO_2_ content of 93% and less than 2% of Fe_2_O_3_. They, however, did not explore the effect of the various leaching variables on the dissolution of iron and titanium systematically, hence little information is available on the reductive leaching of pre-oxidised ilmenite and the full potential of the process has not been optimised.

In this study, the production of synthetic rutile from tin ore beneficiation by-products from Bangka Island, Indonesia contained about 46% of TiO_2_ via pre-oxidation and reductive leaching in hydrochloric acid was investigated. The influence of the pre-oxidation temperature on the type of phases formed during the process was thermodynamically and experimentally investigated. The effect of the leaching variables, namely acid concentration, temperature, pulp density, leaching duration and size fraction of pre-oxidised ilmenite, on the iron and titanium dissolutions were also systematically studied. The experimental matrix was designed using the Taguchi method. The method is based on an orthogonal array which requires a variation of control parameters with different levels for each experiment. It allows the reduction of the number of trials of the multi-parameters experiment, as well as allows the determination of the degree of significance influence and sensitivity of the experimental parameters to the output of the experiment^13–16^. The proposed method in the present study is potentially more economically viable than the current commercial process that uses HCl for the ilmenite leaching, namely the Benelite process. This process involves reduction roasting at higher temperatures (> 850 °C) and leaching at higher temperatures (up to 149 °C) and pressures (up to 3.4 atm) than the proposed method, while using about the same acid concentration in the leaching process.

## Materials dan methods

### Sample preparation and characterisation

**Figure 1:**
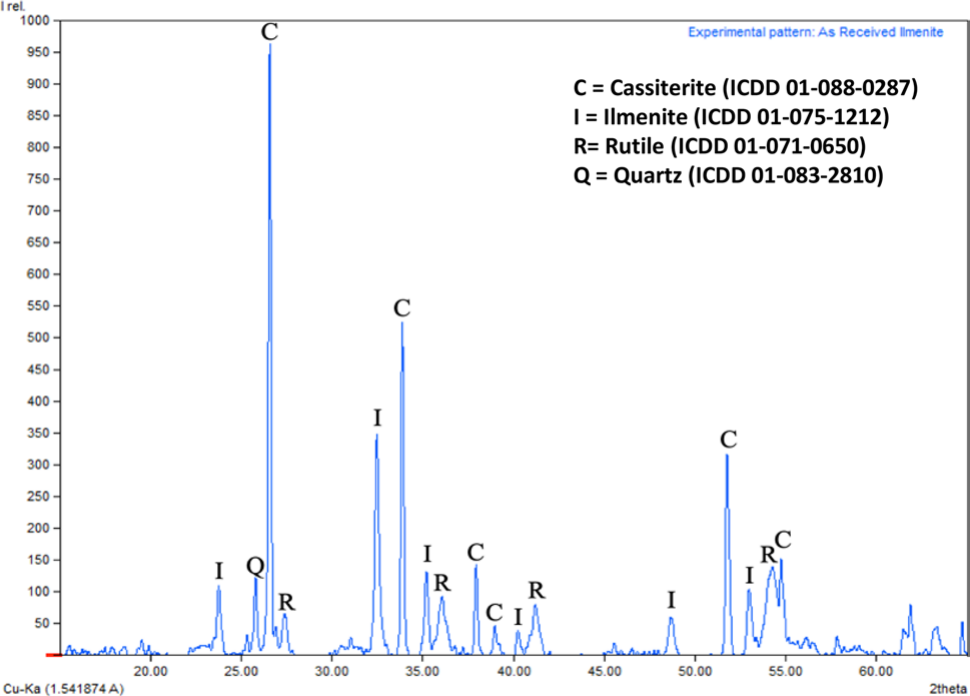
XRD patterns of the ilmenite sample.

As aforementioned, the ilmenite sample used in this study was originally from Bangka Island, Indonesia. Bangka Island is in the east part of Sumatra Island (Latitude: −2° 14′ 60.00″ S, Longitude: 106° 00′ 0.00″ E) and the principal island of the Bangka-Belitung Province in Indonesia. The ilmenite is the product of tin ore beneficiation which consists of a series of gravity, electrostatic and magnetic separations. The ilmenite sample was dried in an electric oven at 110 °C for 24 h, ground in a ball mill and then dry-sieved in a sieve shaker before being used in pre-oxidation experiments. Measurements of the moisture content showed that the sample contains only 0.43% moisture, which indicated that it was already in a dry state. The chemical composition of the ilmenite sample determined by X-ray Fluorescence (XRF, Rigaku Supermini 200, Japan) is shown in Table 1. The ilmenite sample has TiO_2_ and Fe_2_O_3_ contents of 50.9% and 25.4%, respectively with SnO_2_ content of 10.9%. The minerals in the sample were determined by X-ray diffraction analysis (XRD, Rigaku SmartLab, Japan). The chemical composition analysis result with XRF is in accordance with XRD analysis (Fig. 1) which identified cassiterite (SnO_2_) along with ilmenite (FeTiO_3_), rutile (TiO_2_) and quartz (SiO_2_) as the dominant minerals. The particle size distribution of the sample, depicted in Table 2, shows that most of the particle size of the ilmenite sample is in the range of 105–149 microns (–100 to + 140 mesh).

**Table 1:** Chemical composition of the ilmenite sample.

Component	Content (%)
TiO_2_	50.6
Fe_2_O_3_	25.4
SnO_2_	10.9
V_2_O_5_	4.86
MnO	2.64
Y_2_O_3_	0.54
Al_2_O_3_	1.59
Nb_2_O_5_	0.35
SiO_2_	1.32
In_2_O_3_	0.21
SO_3_	0.88
P_2_O_5_	0.43
ZrO_2_	0.12
PbO	0.10
ZnO	0.05

**Table 2:** Particle size distribution of the ilmenite sample.

Size fraction (µm)	Weight (gram)	Percentage
> 149	0.8	0.2
105–149	372.8	93.2
74–105	1.5	0.38
44–74	23.5	5.88
< 44	1.4	0.35
Total	400	100

### Pre-oxidation experiment

The objective of the pre-oxidation of ilmenite concentrate is to convert the ilmenite (FeTiO_3_) into rutile (TiO_2_) and hematite (Fe_2_O_3_). Thermodynamic simulation of the pre-oxidation of ilmenite concentrate was conducted using FactSage 7.2 thermochemical software^17^. Series of experiments were performed using the ilmenite samples each with a mass of 30 g. The samples were pre-oxidized under temperature variations of 700 °C, 800 °C, 900 °C, and 1000 °C in a muffle furnace for 6 h. The furnace was not tightly sealed and the air ingress from the surrounding ensure that the oxidation can completely occur. The products resulting from various pre-oxidation temperatures were analysed by XRD to elucidate the phase alterations that took place at various pre-oxidation temperatures. The optimum condition of the pre-oxidation of ilmenite was achieved when most ilmenite converted into rutile (TiO_2_) and hematite (Fe_2_O_3_), while minimizing the formation of pseudobrookite (Fe_2_TiO_5_).

### Leaching experiment

The pre-oxidized ilmenite at the optimum condition was then treated in reductive leaching experiments using hydrochloric acid and iron powder as reducing agent to selectively dissolve iron while minimizing co-dissolution of titanium and concentrating TiO_2_ in the leach residue. The experimental design of the leaching of pre-oxidized ilmenite was established by Taguchi Method. Series of the leaching experiments were carried out under variations of five variables, namely hydrochloric acid (HCl) concentration, temperature, solid to liquid (S/L) ratio, ilmenite size fraction and duration of leaching. Each variable was varied at four different levels according to the Taguchi Orthogonal Array (OA) design of L16: 4^5^. The variables and their levels of variation are presented in Table 3. Based on the variables and their level of variations, the experimental program was determined, and the detail is presented in Table 4. The experiments were conducted in duplicate for each experimental condition to obtain a high confidence level of the results.

**Table 3:** Experimental variable and its level of variation.

Parameters	Level			
	1	2	3	4
HCl concentration (M)	5	6	7	8
Temperature (°C)	70	80	90	100
S/L ratio	1/5	1/10	1/15	1/20
Ore size fraction	105–149 μm (− 100 + 140#)	74–105 μm (− 140 + 200# )	44–74 μm (− 200 + 325#)	37–44 μm (− 325 + 400#)
Leaching duration (hours)	6	8	10	12

**Table 4:** The experimental plan designed by orthogonal L16: 4^5^ of the Taguchi method.

Exp. number	Parameters and their levels				
	[HCl] (M)	T (°C)	S/L	Size fraction (μm)	Time (h)
1	5	70	1/5	105–149	6
2	5	80	1/10	74–105	8
3	5	90	1/15	44–74	10
4	5	100	1/20	37–44	12
5	6	70	1/10	44–74	12
6	6	80	1/5	37–44	10
7	6	90	1/20	105–149	8
8	6	100	1/15	74–105	6
9	7	70	1/15	37–44	8
10	7	80	1/20	44–74	6
11	7	90	1/5	74–105	12
12	7	100	1/10	105–149	10
13	8	70	1/20	74–105	10
14	8	80	1/15	105–149	12
15	8	90	1/10	37–44	6
16	8	100	1/5	44–74	8

**Figure 2:**
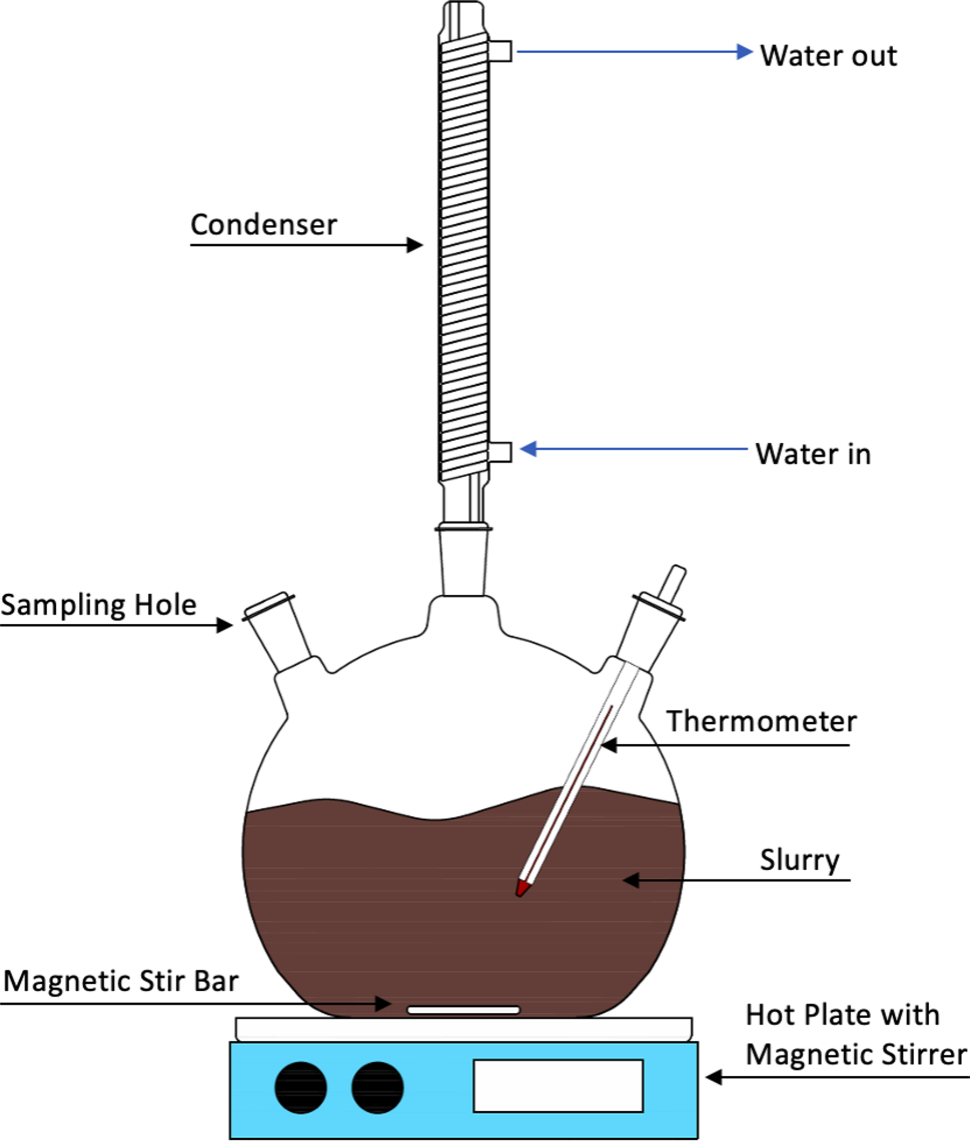
Schematic picture of the leaching experiment.

The leaching experimental set-up is shown schematically in Fig. 2. The leaching experiment was carried out in a 500-mL three-necks flask with a condenser attached to one of the necks to condense the evaporated water, thus a constant volume of the solution during the leaching can be maintained. Agitation of the slurry was done by using a magnetic stirrer which was integrated with a hot plate. The temperature of the solution during the leaching was measured by a thermocouple immersed in the solution. The thermocouple was connected to the hotplate for adjustment of the heating level to keep the leaching temperature at ± 0.5 °C around the targeted temperature. The volume of the initial HCl solution was 200 mL. The stirring speeds during leaching were kept constant at 700 rpm. For each experiment, iron powder as a reducing agent (i.e., reduces Fe^3+^ to Fe^2+^) was added to the solution 20 min after the leaching started with a constant iron/ilmenite mass ratio of 1/5.

Filtration of the slurry by filter paper was carried out upon completion of the leaching experiments. The filtrates were analysed by atomic absorption spectroscopy (AAS, Shimadzu AA-6300, Japan) for measurements of dissolved iron and titanium concentrations, while the solid residues were washed, dried, and digested using concentrated hydrofluoric acid for determining the mass of iron and titanium remained in the leach residue. The solution obtained by the digestion of the leach residue was also analysed by AAS for measurements of iron and titanium concentrations in the leach residue for each experiment. The percentages of extracted iron and titanium were calculated based on the mass ratio of the dissolved metals in pregnant leach solution (PLS) and the sum of the dissolved metals in the PLS and those remaining in the solid residue as formulated by the following equation:

1 $$Extracted\;metal = \frac{{m_{1} }}{{m_{1} + m_{2} }} \times 100\%$$

in which m_1_ is the mass of dissolved metals in PLS (i.e., Ti for the calculation of Ti extraction and Fe for Fe extraction) and m_2_ is the mass of metals remaining in the solid residue. As previously mentioned, the objective of the leaching is to maximise the dissolution of iron, while minimising co-dissolution of titanium, thus the residue which contains a high concentration of TiO_2_ can be obtained.

## Result and discussion

### Thermodynamic simulation of ilmenite pre-oxidation

The thermodynamic aspect of the pre-oxidation of ilmenite was evaluated by performing a calculation of the equilibrium reaction between ilmenite concentrate and air atmosphere (pO_2_ = 0.21 atm) at temperature intervals between 700 and 1000 °C using the FactSage 7.2 thermochemical package. The thermodynamic simulation was performed based on the ilmenite concentrate composition provided in Table 1 and FactSage public database which included FactPS (for pure gas and solids) and FToxid (for solid and liquid oxide solutions). The pseudobrookite solution model in the FactSage public database does not contain a ferric-pseudobrookite (Fe_2_TiO_5_) end member. To approximate a continuous solid solution between the ferric-pseudobrookite and other pseudobrookite end members (FeTi_2_O_5_, Ti_3_O_5_, MnTi_2_O_5_) in the public database, the ferric-pseudobrookite compound was merged into the existing pseudobrookite solution model and was assumed to have ideal interaction with other end-members in the solution.

**Figure 3:**
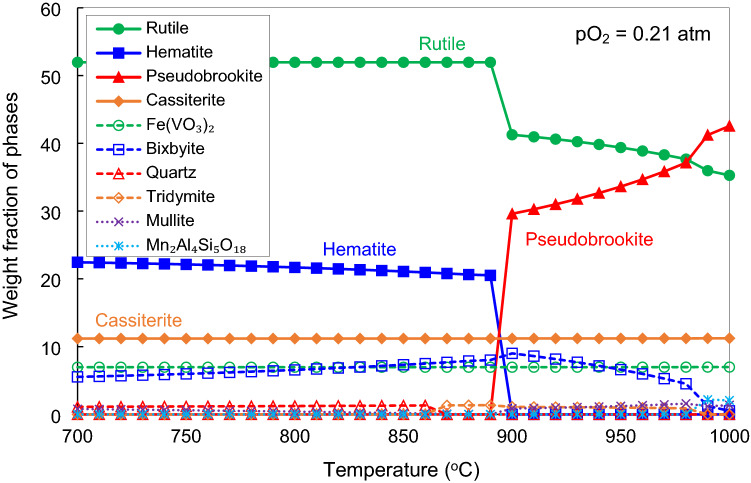
Thermodynamic calculation of the stability of phases as a function of temperature during the pre-oxidation of ilmenite concentrate in air atmosphere.

The result of the equilibrium calculation is summarized in Fig. 3. The figure shows the formation of rutile, hematite, pseudobrookite, cassiterite, Fe(VO_3_)_2_, bixbyite, quartz, tridymite, mullite, and cordierite (Mn_2_Al_4_Si_5_O_18_). In general, rutile, hematite and bixbyite are formed at temperatures below 900 °C. Pseudobrookite starts to form at the expense of rutile, hematite and bixbyite at above 900 °C. Cassiterite and Fe(VO_3_)_2_ pure compounds are thermodynamically stable due to the high tin and vanadium concentrations in the ilmenite concentrate and the absence of tin and vanadium-containing solutions in the database. Fe_2_O_3_ and Mn_2_O_3_ are not completely miscible under an air atmosphere and thus form two immiscible phases of hematite and bixbyite^18^. Quartz transforms into tridymite at around 865 °C, which further reacts with other components at above 900 °C to form mullite or cordierite.

### Determination of optimum temperature of ilmenite pre-oxidation

The pre-oxidation experiments of the ilmenite concentrate were performed at 700 °C, 800 °C, 900 °C, and 1000 °C in a muffle furnace for 6 h. The objective of the pre-oxidation process is to convert the ilmenite into rutile (TiO_2_) and hematite (Fe_2_O_3_), while minimizing the formation of pseudobrookite (Fe_2_TiO_5_). At the leaching stage, the iron in the hematite is expected to be dissolved as much as possible, with minimum co-dissolution of titanium from the rutile. Through this mechanism, synthetic rutile can be concentrated in the leach residue.

**Figure 4:**
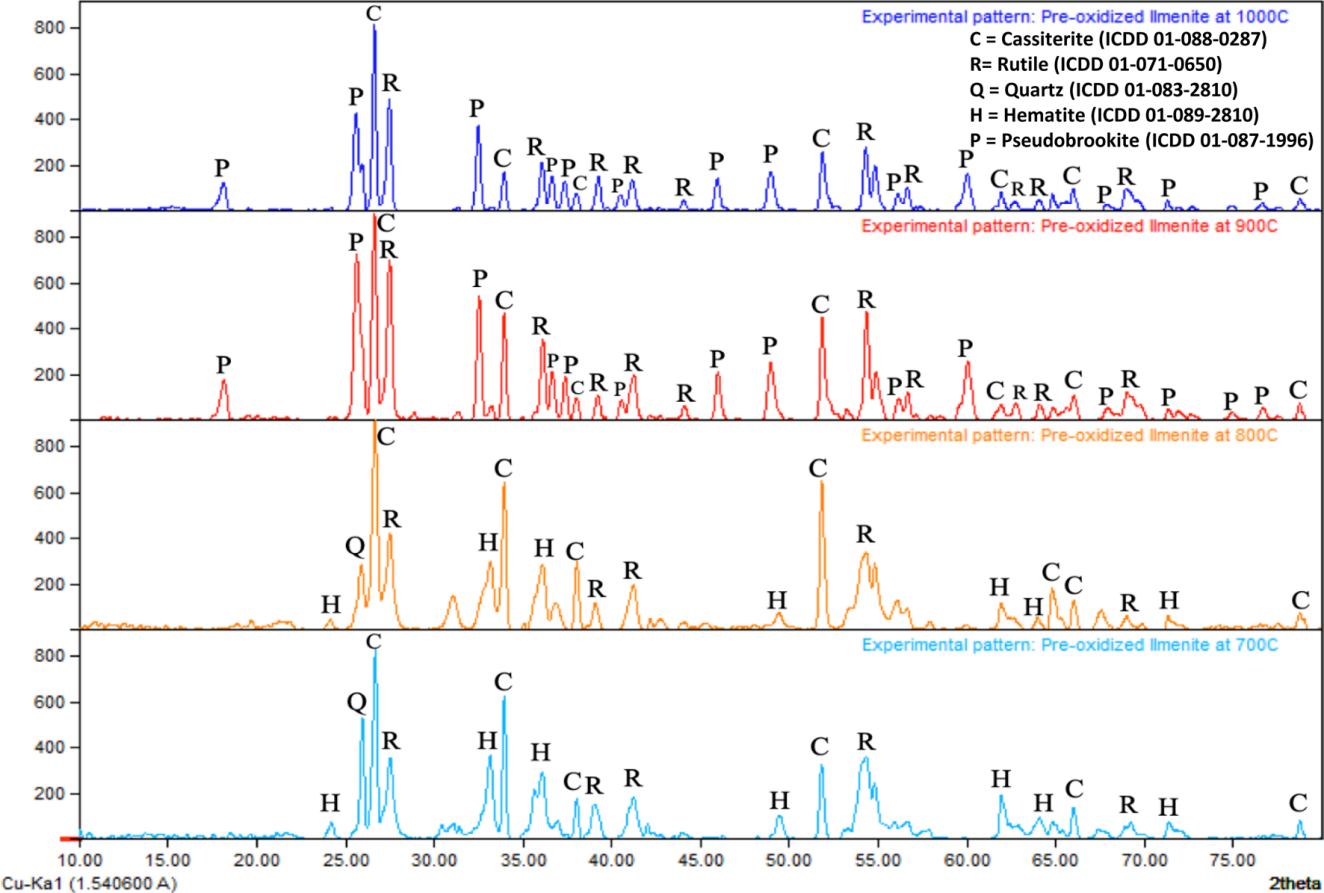
XRD patterns of the products of ilmenite pre-oxidation at various temperatures.

XRD spectra of the products of pre-oxidation at various temperatures are depicted in Fig. 4. According to the XRD analysis of the pre-oxidation product, at temperatures of 700 °C dan 800 °C, ilmenites (FeTiO_3_) were mostly converted to rutile (TiO_2_) and hematite (Fe_2_O_3_), while at 900 °C dan 1000 °C a new phase called pseudobrookite (Fe_2_TiO_5_) was also formed along with rutile. Cassiterite (SnO_2_) was detected in the oxidised products at the entire temperature variations. However, the peak intensity related to cassiterite is diminished in the products formed at 900 and 1000 °C. Meanwhile, the peak intensities for quartz (SiO_2_) were diminished completely by oxidation temperatures higher than 800 °C. In general, the experimental results in Fig. 4 are in agreement with the thermodynamic simulation results as shown in Fig. 3. However, several phases which supposed to be thermodynamically stable were not visible in the XRD pattern which may be due to uncertainty in the reported concentrations of minor components in the ilmenite concentrate, low concentrations of minor phases in the pre-oxidized samples, overlapping XRD peaks in the pre-oxidized samples, metastable condition resulted by the low kinetic in the solid-state reaction, or unoptimized database used in the thermodynamic calculation.

Vásquez and Molina^12^, who also studied pre-oxidation of ilmenite, reported that the hematite formed during oxidation is concentrated on the outer layer of the oxidized particle, while rutile is enriched in the inner layer. This was suggested to be associated with the diffusion of iron cation to the region which has high oxygen potential leading to the formation of hematite at the surface and purer rutile at the core of the particle^19^. The enrichment of hematite at the surface of the particle would facilitate faster iron dissolution under reductive conditions and on the other hand, titanium that is contained in rutile at the inner parts of the particle will be less amenable to acid dissolution and will be concentrated in the leach residue. Hence, the phenomenon that takes place during the pre-oxidation allows a better leaching selectivity of iron toward titanium at subsequent reductive acid leaching of the pre-oxidized ilmenite. The oxidation of ilmenite to hematite and rutile occurs through the following reaction^20^:

2 $${\text{2FeTiO}}_{{{3}({\text{s}})}} + \raise.5ex\hbox{$\scriptstyle 1$}\kern-.1em/ \kern-.15em\lower.25ex\hbox{$\scriptstyle 2$} {\text{O}}_{{{2}({\text{g}})}} = {\text{Fe}}_{{2}} {\text{O}}_{{{3}({\text{s}})}} + {\text{2TiO}}_{{{2}({\text{s}})}}$$

Meanwhile, the formation of pseudobrookite (Fe_2_TiO_5_) takes place through the following reaction:

3 $${\text{2FeTiO}}_{{\text{3(S)}}} + \raise.5ex\hbox{$\scriptstyle 1$}\kern-.1em/ \kern-.15em\lower.25ex\hbox{$\scriptstyle 2$} {\text{O}}_{{\text{2(g)}}} = {\text{TiO}}_{{\text{2(s)}}} + {\text{Fe}}_{{2}} {\text{TiO}}_{{\text{5(s)}}}$$

The formation of the pseudobrookite phase in the pre-oxidation products at 900 and 1000 °C are in agreement with the findings of Vásquez and Molina^12^ and Zhang and Ostrovski^20^. It was reported that the presence of pseudobrookite in the pre-oxidation product of ilmenite slows down the dissolution of iron and reduces total dissolved iron in the subsequent leaching stage^12, 21^. Noticeably, the peaks of XRD spectra associated with hematite at 700 °C are stronger than those formed at 800 °C, which is in agreement with the findings of Vásquez and Molina^12^.

Based on the thermodynamic simulation and XRD analysis results of the pre-oxidation of ilmenite at various temperatures, the temperature of 700 °C was considered as the optimum temperature since it can produce the best conversion of ilmenite to hematite and rutile without the formation of pseudobrookite.

### Effect of leaching variables on extracted iron and titanium analysed by ANOVA

Results of leaching experiments are shown in Table 5. The leaching experiment results showed that extracted iron was in the range of 94.24–97.54%, while co-dissolved titanium varied from 3.31 to 23.15%. Analysis of variance (ANOVA) was used to determine the level of influence of each leaching variable on the extraction levels of iron and titanium. This analysis involved the calculation of the parameters of the sum of square (SS), mean square (MS), and F-distribution. The value of standard F-distribution for iron and titanium dissolutions was then obtained from the F-distribution table using α = 0.05, df_1_ = 3, and df_2_ = 16, which is equal to 3.24. By comparing the value of the F-distribution from each variable to the standard F-distribution, the significance of the influence of each variable on the extractions of Fe and Ti can be assessed. The results of ANOVA which shows the influence of each variable represented by the contribution percentage of the variable on the extracted Fe and Ti are presented in Tables 6 and 7, respectively. By using a standard F-distribution value of 3.24, it can be concluded that HCl concentration, temperature, S/L ratio, and ilmenite particle size distribution have a significant influence on the extraction of iron. The temperature was found to have the highest influence on the iron extraction (42.98%), followed by S/L ratio (30.37%), acid concentration (15.81%) and ilmenite particle size distribution (4.21%). Generally, the dissolution rate of metals in an aqueous solution is enhanced when the leaching temperature is higher. It was also indicated that the leaching of ilmenite in hydrochloric acid is mass-transfer controlled, therefore, the temperature is one of the most decisive parameters that affect the diffusion rate of reacting species and determines the extracted iron and titanium at a certain duration of the leaching^21^.

**Table 5:** Results of leaching experiment of pre-oxidized ilmenite.

Experiment no	Extracted Fe (%)			Ti co-dissolution (%)		
	Duplicate I	Duplicate II	Mean	Duplicate I	Duplicate II	Mean
1	94.52	93.96	94.24	2.87	3.75	3.31
2	96.05	96.29	96.17	7.49	8.23	7.86
3	96.82	96.95	96.89	11.30	14.94	13.12
4	97.41	97.04	97.22	16.68	16.37	16.53
5	95.95	96.00	95.97	12.67	11.97	12.32
6	96.64	97.01	96.82	8.45	9.09	8.77
7	97.74	97.34	97.54	22.44	21.91	22.18
8	97.20	97.54	97.37	10.76	11.61	11.19
9	96.50	95.77	96.13	18.36	17.50	17.93
10	98.46	97.98	98.22	19.23	18.30	18.76
11	96.20	96.64	96.42	8.09	8.74	8.42
12	97.36	96.51	96.94	16.65	15.88	16.26
13	96.45	96.73	96.59	23.92	22.38	23.15
14	97.18	97.35	97.27	14.41	16.41	15.41
15	97.36	97.22	97.29	20.33	21.52	20.92
16	96.44	97.01	96.72	10.10	13.76	11.93

**Table 6:** Result of the analysis of variance for the dissolution of iron.

Parameters	Degree of Freedom	Sum of squares	Mean square	F distribution	Contribution (%)
HCl concentration (M)	3	3.96	1.32	13.8	15.81
Temperature (°C)	3	10.76	3.59	37.53	42.98
S/L ratio	3	7.6	2.53	26.52	30.37
Particle size distribution	3	1.05	0.35	3.67	4.21
Leaching duration (hours)	3	0.13	0.04	0.46	0.53
*Error*	16	1.53	0.1	1	6.11
Total	31	25.03			100

**Table 7:** Result of the analysis of variance for the dissolution of titanium.

Parameters	Degree of freedom	Sum of squares	Mean square	F distribution	Contribution (%)
HCl concentration (M)	3	247.66	82.55	65.35	25.51
Temperature (°C)	3	48.97	16.32	12.92	5.04
S/L ratio	3	581.04	193.68	153.31	59.84
Particle size distribution	3	46.35	15.45	12.23	4.77
Leaching duration (hours)	3	26.79	8.93	7.07	2.76
*Error*	16	20.21	1.26	1	2.08
Total	31	971.02			100

Similarly, all variables also exhibited a significant influence on the dissolution of titanium. Different to the case for iron dissolution, the S/L ratio was found to have the highest influence on the titanium extraction (59.84%), followed by acid concentration (25.51%), temperature (5.04%), ilmenite particle size distribution (4.77%) and duration of the leaching (2.76%). As has been previously mentioned, during the pre-oxidation stage, Fe_2_O_3_ is concentrated on the outer layer of the oxidized particle, while TiO_2_ is enriched in the inner layer of the oxidized particle. Titanium present in rutile at the inner parts of the particle is less amenable to acid dissolution in comparison to iron which is present at the outer layer. At a smaller solid/liquid ratio, more HCl is available in the solution which in turn will have a higher probability to diffuses to the inner layer and reacting with TiO_2_. In this regard, the variable S/L ratio eventually gives the highest influence on the dissolution of titanium-based on the experimental design by Taguchi L16: 4^5^ and data analysis by ANOVA.

### Signal to noise ratio analysis and the optimum condition evaluation for the iron and titanium extractions

**Figure 5:**
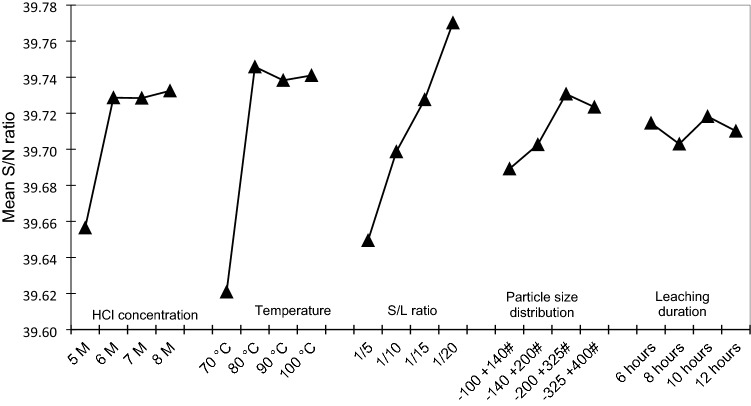
Profile of S/N ratio at various levels of the experimental variables which respect iron extraction percentage.

**Figure 6:**
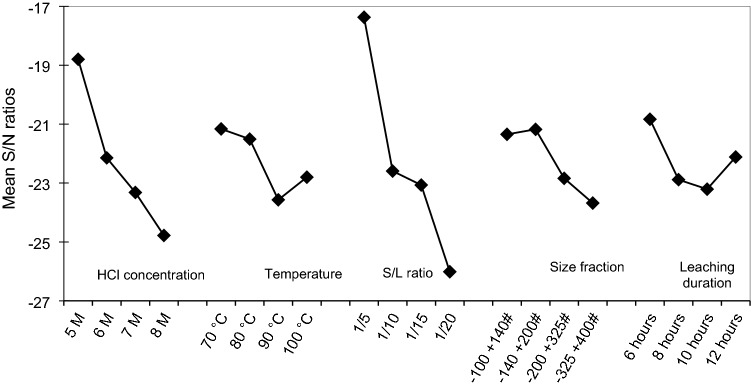
Profile of S/N ratio at various levels of the experimental variables which respect to titanium co-extraction percentage.

A statistical correlation between the experimental factors (variables) and the experimental results (outputs) was further evaluated. Signal-to-noise (S/N) ratios, which is a measure of the effect of the experimental variations on the outputs of the experiment by minimizing the effect of noise factors, for each of the control parameters were calculated. The degrees of influence of variables, represented by the parameter of signal to noise (S/N) ratios, to the experimental outputs, i.e., the dissolutions of iron and titanium, are given in Figs. 5 and 6, respectively. Since the target of the leaching process is to maximize the dissolution of iron and minimize the co-dissolution of titanium, the desired condition for the iron dissolution is indicated by the highest S/N value in Fig. 5, while the desired condition for titanium dissolution is indicated by the lowest S/N value in Fig. 6. Based on the profiles of S/N ratios for Fe and Ti extractions versus leaching variables, the condition with the highest S/N ratio for iron extraction and lowest S/N ratio for titanium co-extraction is considered at HCl concentration of 8 M, the temperature of 90 °C, S/L ratio 1/20 g/ml, the particle size distribution of 44–74 µm, and leaching duration of 10 h.

The optimum condition was further evaluated through simple mathematical models that describe the extractions of Fe and Ti as a function of variables of the leaching process based on the experimental results in Table 5. The models were established by using Minitab software. The models suggested that the combined optimum condition for high iron extraction and low titanium co-extraction is at an HCl concentration of 6 M, a temperature of 80 °C, an S/L ratio of 1/20 g/ml, and the particle size distribution of 44–74 µm, and leaching duration of 6 h. HCl concentration of 6 M was considered as an optimum level to balance the two objectives (i.e., maximizing Fe extraction, while minimizing co-extracted Ti). The increase of HCl concentration above 6 M did not significantly promote the extraction percentage of iron which is indicated by relatively flat S/N by the increase in HCl concentration from 6 to 8 M. Ramadan et al.^22^ stated that the dissolution of iron in ilmenite linearly increases to a concentration of 20% HCl (around 6.5 M) and the further increase of HCl concentration beyond this level did not significantly affect the extracted iron percentage. On the other hand, higher HCl concentration also promotes the dissolution of titanium, thereby increasing the loss of titanium which is desirably concentrated in the leach residue as synthetic rutile.

### Leaching experiment at optimum condition

**Figure 7:**
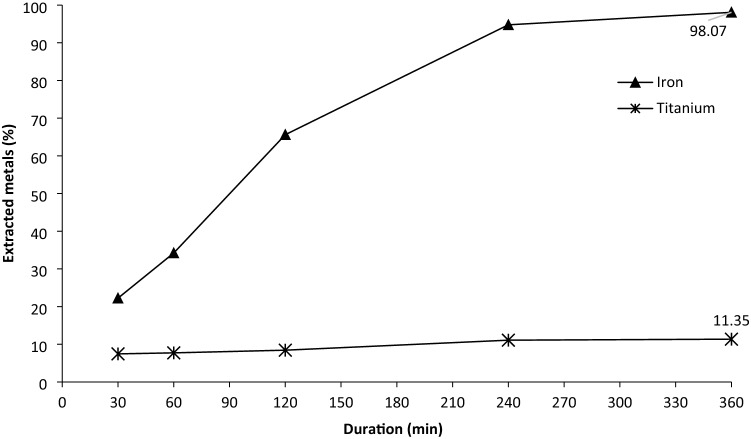
Profiles of extracted iron and co-extracted titanium during the leaching of pre-oxidised ilmenite at the optimum condition: [HCl] = 6 M, T = 80 °C, S/L = 1/20 g/ml, size fraction = 44–74 µm, duration = 6 h.

The extracted Fe and Ti at the optimum condition from the mathematical models were predicted to be 98.22% and 17.03%, respectively. To verify the predicted Fe and Ti extractions, a leaching experiment was carried out at the optimum condition, namely at 6 M HCl concentration, the temperature of 80 °C, S/L 1/20 (g/mL), ilmenite size fraction of 44–74 µm, and leaching duration of 6 h with iron powder ratio to ilmenite of 1/5 added in the solution and stirring speed 700 rpm. During the leaching experiment, slurry samples were collected at a pre-determined volume and time interval (i.e., 30, 60, 120, 240 and 360 min). The slurry samples were filtered through Whatman filter paper, and the filtrates were subsequently analysed by AAS to determine the concentrations of dissolved Fe and Ti. The result of the leaching experiment at optimum condition is shown in Fig. 7.

Based on the experimental results presented in Fig. 7, the highest iron extraction level of 98.07% was obtained after 360 min of leaching. This value was very close to that calculated by the mathematical model for the iron extraction (98.22%). Meanwhile, the co-extraction of titanium after 360 min was 11.35% which is 5.68% lower than that calculated by the mathematical model for the titanium extraction (i.e., 17.03%). This difference is suggested to be associated with the different extents of titanium hydrolysis which cannot be controlled during the experiments.

The dominant reaction taking place during the leaching of the pre-oxidised ilmenite is the dissolution of iron. The dissolution kinetics of iron from the pre-oxidised ilmenite at the optimum condition (Fig. 7) has been analysed to determine its controlling step. The pre-oxidised particles are assumed to have a relatively uniform particle size, dense structure, and spherical geometry. In the case where hematite occupied the outer layer of the pre-oxidised particle, there was no barrier between the unreacted hematite and the leaching medium. In addition, the external radius of the pre-oxidised particle gradually decreased during the dissolution of iron. This situation can be evaluated using the shrinking core models for the shrinking sphere^23^. Two rate-controlling steps were considered, i.e., diffusion of reactant from the bulk fluid to the solid surface through the film layer and chemical reaction on the solid surface. The kinetic analysis based on the selected models indicated that the dissolution kinetic of iron in this study was controlled by the chemical reaction on the solid surface.

Based on the data of iron and titanium extractions, iron extraction relative to titanium was determined by comparing the extraction percentage of iron with the sum of iron extraction percentage and titanium extraction percentage. The Fe extraction relative to Ti was 0.90 which indicates that co-extracted Ti was relatively low and most of the titanium was concentrated in the leaching residue.

### Mechanism of the leaching of pre-oxidized ilmenite in hydrochloric acid in the presence of iron powder

Prior to the pre-oxidation process of ilmenite ore, Fe exists as Fe(II) or FeO as well as FeTiO_3_ and distributed evenly throughout the ilmenite ore particle. The dissolution of iron and titanium during direct leaching of ilmenite without pre-oxidation treatment takes place simultaneously through the following reaction^10^:

4 $${\text{FeTiO}}_{{{3}({\text{s}})}} + {\text{4HCl}}_{{({\text{aq}})}} = {\text{FeCl}}_{{{2}({\text{aq}})}} + {\text{TiOCl}}_{{{2}({\text{aq}})}} + {\text{2H}}_{{2}} {\text{O}}_{{({\text{l}})}}$$

Basically, both of iron and titanium are dissolved and consume the leaching agent, although part of dissolved titanium is slowly hydrolysed and releases hydrochloric acid according to the following reaction:

5 $${\text{TiOCl}}_{{{2}({\text{aq}})}} + {\text{H}}_{{2}} {\text{O}}_{{({\text{l}})}} = {\text{TiO}}_{{{2}({\text{s}})}} + {\text{2HCl}}_{{({\text{l}})}}$$

As previously mentioned, during the pre-oxidation stage of ilmenite at a temperature range of 700–1000 °C, the Fe cations diffuse to the outer layer of ilmenite particle and form hematite which will be more prone to the leaching by HCl. The dissolution rate of Fe during the leaching of pre-oxidised ilmenite is fast in the first few hours of the leaching which slows down by the longer time of the leaching^21^.

Since rutile occupies the inner part of the pre-oxidized ilmenite, the leaching of Ti is minimized and controlled by the diffusion of reactant and reaction product through the solid particle^21^. In the absence of a reducing agent, the leaching of iron from hematite in pre-oxidized ilmenite by hydrochloric acid occurs through the following reaction^10^:

6 $${\text{Fe}}_{{2}} {\text{O}}_{{{3}({\text{s}})}} + {\text{6HCl}}_{{({\text{aq}})}} = {\text{2FeCl}}_{{{3}({\text{aq}})}} + {\text{3H}}_{{2}} {\text{O}}_{{({\text{l}})}}$$

In the presence of Fe powder as a reducing agent, iron(II) chloride and H_2_ gas will be formed as the product of the reaction between Fe powder and HCl (Reaction 7). Iron(III) ions released from the leaching of hematite (Reaction 6) will be readily reduced by the hydrogen gas (H_2_) and Fe powder simultaneously (Reactions 8 and 9). Consequently, most of the Fe(III) ions will be reduced to Fe(II) ions which are more soluble in HCl solution compared to Fe(III) ions^10^.

7 $${\text{Fe}}_{{({\text{s}})}} + {\text{2HCl}}_{{({\text{aq}})}} = {\text{FeCl}}_{{{2}({\text{aq}})}} + {\text{H}}_{{{2}({\text{g}})}}$$

8 $${\text{2FeCl}}_{{{3}({\text{aq}})}} + {\text{H}}_{{{2}({\text{g}})}} = {\text{2FeCl}}_{{{2}({\text{aq}})}} + {\text{2HCl}}_{{({\text{aq}})}}$$

9 $${\text{2FeCl}}_{{{3}({\text{aq}})}} + {\text{Fe}}_{{({\text{s}})}} = {\text{3FeCl}}_{{{2}({\text{aq}})}}$$

### Analysis of the leach residues

**Figure 8:**
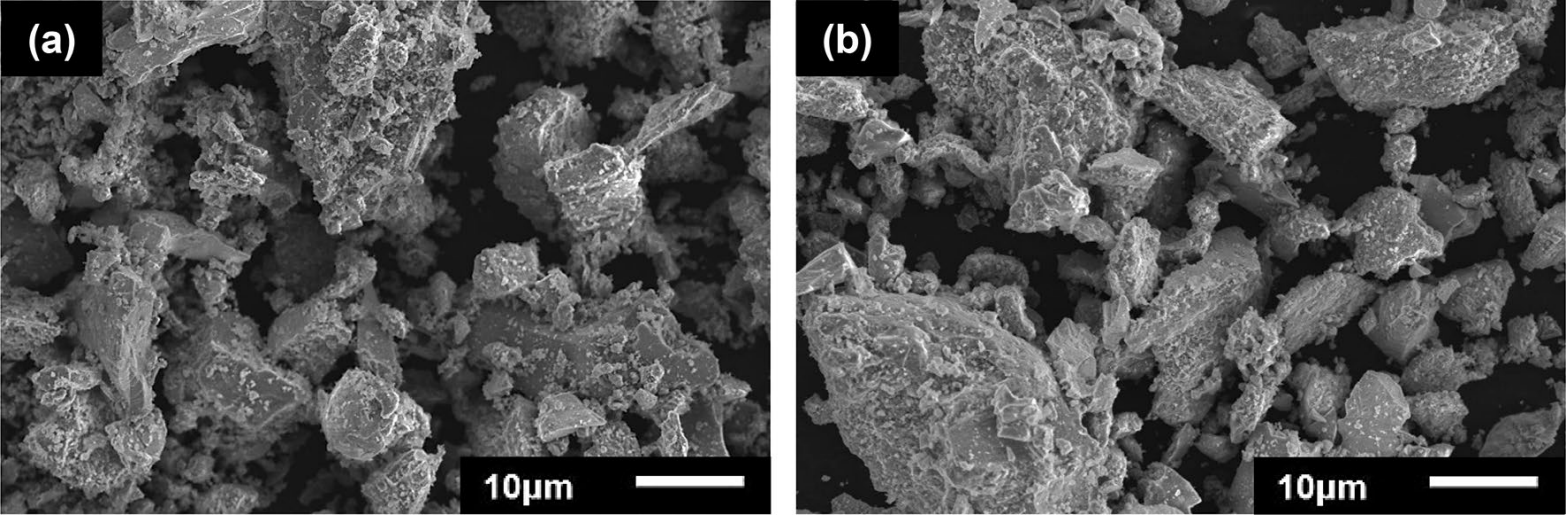
Morphological structures of (**a**) ilmenite; and (**b**) leaching residue.

**Figure 9:**
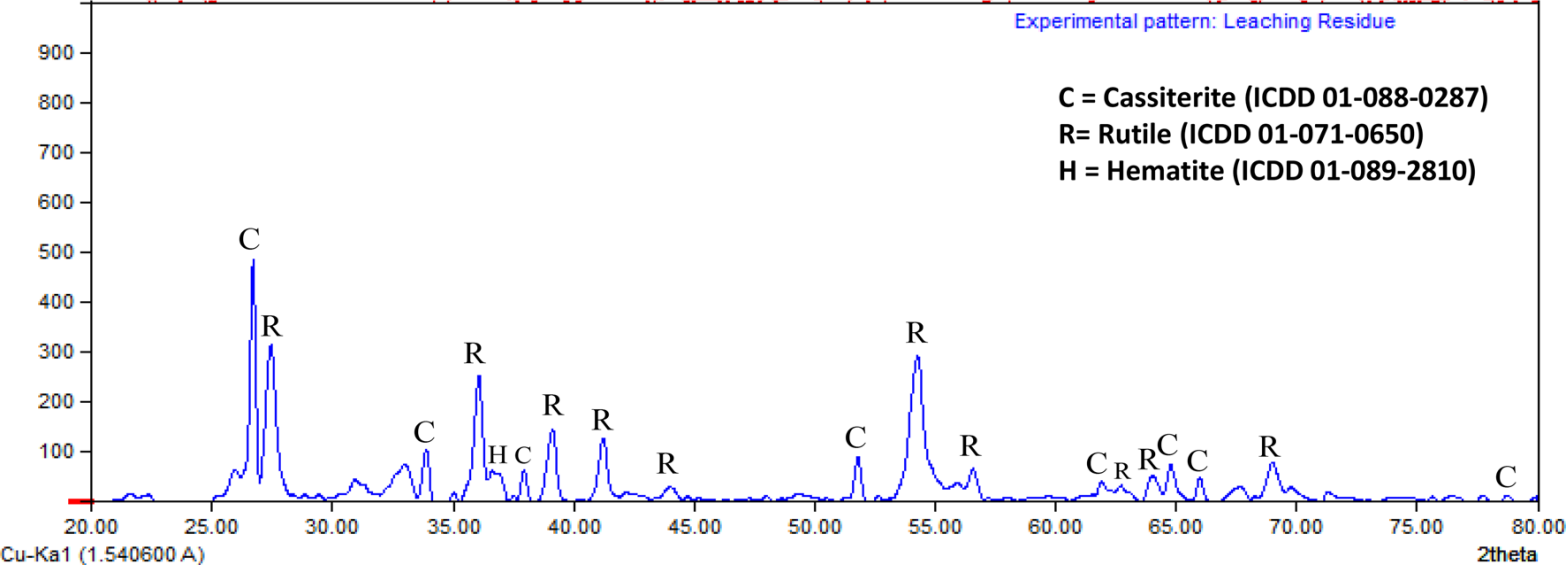
XRD patterns of the leaching residue.

The residue obtained from the leaching experiment of pre-oxidized ilmenite at optimum condition (i.e., HCl concentration of 6 M, temperature 80 °C, solid/liquid ratio 1/20, size fraction 44–74 µm, and 6 h of leaching duration) was analysed by scanning electron microscope (SEM, JEOL JSM-6510A, Japan), XRD and XRF. The SEM images of the surface morphologies of ilmenite and leaching residue particles are shown in Fig. 8. The ilmenite sample consisted of particles with a wide range of sizes from a few microns up to 149 microns, similarly, the leaching residue had a broad particle size distribution. Based on the SEM examination, the larger ilmenite particles had relatively smooth surface morphology while the larger leaching residue particles had rougher surface morphology which is possibly due to reaction with the HCl. The XRD analysis result of the leaching residue is shown in Fig. 9. The XRD analysis result shows that the leaching residue consists of mostly rutile and cassiterite and indicates that most of the hematite was effectively dissolved during the leaching process. The sharp peak of rutile in the XRD pattern of leaching residue is related to the rutile’s low solubility in the leaching stage. The XRD spectrum shows only 1 peak of hematite which indicates a minor amount of the hematite in the leaching residue. The chemical composition of the leaching residue was determined by XRF analysis, and the result is shown in Table 8. The leaching residue contains 92.6% TiO_2_ and 2.9% Fe_2_O_3_. Other metal compounds which have remarkable percentage in the leaching residue are SnO_2_ (2.5%), V_2_O_5_ (0.9%), MnO_2_ (0.3%) and SiO_2_ (0.3%). The SnO_2_ is present in the leaching residue since the ilmenite was a by-product of the tin ore beneficiation process.

**Table 8:** Result of XRF analysis of the leaching residue.

Compound	Concentration (%)
TiO_2_	92.6
Fe_2_O_3_	2.9
SnO_2_	2.5
V_2_O_5_	0.9
MnO_2_	0.3
SiO_2_	0.3
Al_2_O_3_	0.1
Nb_2_O_5_	0.1
Y_2_O_3_	0.1
WO_3_	0.1
P_2_O_5_	0.1
Total	100

The residue obtained from the leaching of pre-oxidised ilmenite ore under the optimum condition can be considered as synthetic grade rutile which can be utilized for the manufacturing of pure TiO_2_ pigment. The concentration of TiO_2_ of 92.6% is remarkably higher than the rutile commonly used for pigment manufacturing in Indonesia which has a minimum grade of 80%. The presence of minor amounts of Fe_2_O_3_ and SnO_2_, as well as other trace metal compounds, (i.e., V, Mn, Al, Si, Nb, Y, W and P) in the rutile produced in the present work, are not expected to interfere its application.

## Conclusion

In this study, the production of synthetic rutile from ilmenite as a by-product from tin ore beneficiation through pre-oxidation and reductive leaching of the pre-oxidized ilmenite in hydrochloric acid is discussed. The objective of the pre-oxidation of ilmenite concentrate is to convert the ilmenite into rutile (TiO_2_) and hematite (Fe_2_O_3_), while minimizing the formation of pseudobrookite (Fe_2_TiO_5_). At the leaching stage, the iron in the hematite is expected to be dissolved as much as possible, with minimum co-dissolution of titanium from the rutile. Using this method, synthetic rutile can be concentrated in the leach residue. Thermodynamic evaluation and experimental investigation indicated that the optimum pre-oxidation temperature was 700 °C, which converted ilmenite to hematite and rutile. Temperature and solid/liquid ratio (S/L) were found to be the most influential leaching parameters for the dissolutions of iron and titanium. The optimum conditions for maximizing iron solubility, while minimizing titanium dissolution were obtained at 80 °C, HCl = 6 M, S/L ratio 1/20 g/mL, ore particle size distribution 44–74 µm, and leaching duration of 6 h. Leaching experiments carried out under these conditions resulted in iron extraction of 98.07% and titanium co-extraction of 11.35%. The leaching residue contains 92.6% rutile, 2.9% hematite, and 2.5% cassiterite which can be classified as synthetic grade rutile. The investigation results show the utilization prospect of ilmenite as a by-product from tin ore beneficiation for producing synthetic rutile.

## References

[1] Zglinicki K, Szamałek K, Wołkowicz S (2021). Critical minerals from post-processing tailing. A case study from Bangka Island. Indonesia. Minerals.

[2] Braun JH, Baidins A, Marganski RE (1992). TiO_2_ pigment technology: a review. Prog. Org. Coat..

[3] Middlemas S, Fang ZZ, Fan P (2013). A new method for production of titanium dioxide pigment. Hydrometallurgy.

[4] Rosenbaum JB (1982). Titanium technology trends. JOM.

[5] Zhang W, Zhu Z, Cheng CY (2011). A literature review of titanium metallurgical processes. Hydrometallurgy.

[6] Becher, R., Canning, R., Goodheart, B. & Uusna, S. A new process for upgrading ilmenitic mineral sands. in 21–44 (1965).

[7] Chen, J. Beneficiation of titaniferous ores (1974).

[8] Sinha, H. MURSO process for producing rutile substitute. in *Titanium Science and Technology* 233–245 (Springer, 1973).

[9] Walpole, E. & Winter, J. The Austpac ERMS and EARS processes for the manufacture of high-grade synthetic rutile by the hydrochloric acid leaching of ilmenite (Citeseer, 2002).

[10] Mahmoud M, Afifi A, Ibrahim I (2004). Reductive leaching of ilmenite ore in hydrochloric acid for preparation of synthetic rutile. Hydrometallurgy.

[11] Lasheen T (2005). Chemical beneficiation of Rosetta ilmenite by direct reduction leaching. Hydrometallurgy.

[12] Vásquez R, Molina A (2012). Effects of thermal preoxidation on reductive leaching of ilmenite. Miner. Eng..

[13] Moghaddam J, Sarrafmamoory R, Abdollahy M, Yamini Y (2006). Purification of zinc ammoniacal leaching solution by cementation: determination of optimum process conditions with experimental design by Taguchi’s method. Sep. Purif. Technol..

[14] Mondal S, Paul B, Kumar V, Singh D, Chakravartty J (2015). Parametric optimization for leaching of cobalt from Sukinda ore of lateritic origin: a Taguchi approach. Sep. Purif. Technol..

[15] Ni’am AC, Wang Y-F, Chen S-W, You S-J (2019). Recovery of rare earth elements from waste permanent magnet (WPMs) via selective leaching using the Taguchi method. J. Taiwan Inst. Chem. Eng..

[16] Scaffaro R, Sutera F, Lopresti F (2017). Using Taguchi method for the optimization of processing variables to prepare porous scaffolds by combined melt mixing/particulate leaching. Mater. Des..

[17] Bale CW (2016). Reprint of: FactSage thermochemical software and databases, 2010–2016. Calphad.

[18] Kang Y-B, Jung I-H (2016). Thermodynamic modelling of oxide phases in the Fe–Mn–O system. J. Phys. Chem. Solids.

[19] Martin M (2003). Materials in thermodynamic potential gradients. Pure Appl. Chem..

[20] Zhang G, Ostrovski O (2002). Effect of preoxidation and sintering on properties of ilmenite concentrates. Int. J. Miner. Proc..

[21] Vasquez, R. & Molina, A. Leaching of ilmenite and pre-oxidized ilmenite in hydrochloric acid to obtain high grade titanium dioxide (2008).

[22] Ramadan A, Farghaly M, Fathy W, Ahmed M (2016). Leaching and kinetics studies on processing of Abu-Ghalaga ilmenite ore. Int. Res. J. Eng. Tech..

[23] Levenspiel O (1999). Chemical Reaction Engineering.

